# Supplementary material for: Efficacy and Safety of Habitual Consumption of a Food Supplement Containing Miraculin in Malnourished Cancer Patients: The CLINMIR Pilot Study
Source: Nutrients. 2024 Jun 17;16(12):1905. doi: 10.3390/nu16121905 (PMC11207068; doi:10.3390/nu16121905)
Supplement: Supplementary file 1 [file nutrients-16-01905-s001.zip › nutrients-3047083-supplementary.pdf]

nutrients

MDPI

Article (Supplementary Materials)

Efficacy and Safety of Habitual Consumption of a Food Supplement Containing Miraculin in Malnourished Cancer Patients: The CLINMIR Pilot Study

Bricia López-Plaza [1,2,*], Ana Isabel Álvarez-Mercado [3,4,5], Lucía Arcos-Castellanos [1], Julio Plaza-Diaz [4,6,7], Francisco Javier Ruiz-Ojeda [4,5,6,8], Marco Brandimonte-Hernández [5,6], Jaime Feliú-Batlle [9,10,11], Thomas Hummel [12], Ángel Gil [4,5,6,8,†] and Samara Palma-Milla [1,11,13,†]

[1] Food, Nutrition and Health Platform, Hospital La Paz Institute for Health Research (IdiPAZ), 28046 Madrid, Spain; lucia.arcos.castellanos@idipaz.es (L.A.-C.); samara.palma@salud.madrid.org (S.P.-M.)
[2] Medicine Department, Faculty of Medicine, Complutense University of Madrid, Plaza de Ramón y Cajal, s/n, 28040 Madrid, Spain
[3] Department of Pharmacology, University of Granada, 18071 Granada, Spain; alvarezmercado@ugr.es
[4] Instituto de Investigación Biosanitaria ibs.GRANADA, Complejo Hospitalario Universitario de Granada, 18014 Granada, Spain; jrplaza@ugr.es (J.P.-D.); fruizojeda@ugr.es (F.J.R.-O.); agil@ugr.es (Á.G.)
[5] Institute of Nutrition and Food Technology "José Mataix", Centre of Biomedical Research, University of Granada, Avda. del Conocimiento s/n, Armilla, 18016 Granada, Spain; mbrandimonte@ugr.es
[6] Department of Biochemistry and Molecular Biology II, University of Granada, 18071 Granada, Spain
[7] Children's Hospital of Eastern Ontario Research Institute, Ottawa, ON K1H 8L1, Canada
[8] CIBEROBN (CIBER Physiopathology of Obesity and Nutrition), Instituto de Salud Carlos III, 28029 Madrid, Spain
[9] Oncology Department, Hospital La Paz Institute for Health Research—IdiPAZ, Hospital Universitario La Paz, 28029 Madrid, Spain; jaime.feliu@salud.madrid.org
[10] CIBERONC (CIBER Cancer), Instituto de Salud Carlos III, 28029 Madrid, Spain
[11] Medicine Department, Faculty of Medicine, Autonomous University of Madrid, Arzobispo Morcillo 4, 28029 Madrid, Spain
[12] Smell & Taste Clinic, Department of Otorhinolaryngology, Technische Universität Dresden, Fetscherstraße 74, 01307 Dresden, Germany; thomas.hummel@tu-dresden.de
[13] Nutrition Department, Hospital University La Paz, 28046 Madrid, Spain
* Correspondence: bricia.plaza@idipaz.es

Table S1. Perception of food consumption depending on treatment

		Standard dose of DMB			High dose of DMB			Placebo			P-value		
		1 month	2 months	3 months	1 month	2 months	3 months	1 month	2 months	3 months	1 month	2 months	3 months
Eat the same	Yes (%)	66.7	85.7	50	85.7	50	25	50	50	50	0.348	0.298	0.683
	No (%)	33.3	14.3	50	14.3	50	75	50	50	50			
Eat less	Yes (%)	22.2	0	0	0	33.3	50	0	0	0	0.204	0.089	0.032
	No (%)	77.8	100	100,00	100	66.7	50	100	100	100			
Eat more	Yes (%)	11.1	14.3	50,00	14.3	16.7	25	50	50	50	0.172	0.280	0.683
	No (%)	88.9	85.7	50,00	85.7	83.3	75	50	50	50			

Table S2. Nutritional status depending on treatment (%)

		Standard dose of DMB		High dose of DMB		Placebo		p-value	
		Baseline	3 months	Baseline	3 months	Baseline	3 months	Baseline	3 months
Weight lost percentage	No	0	87.5	0	83.3	0	100		
	5 – 10	70	12.5	81.8	16.7	70	0	0.772	0.556
	>10	30	0	18.2	0	30	0		
GLIM Criteria Malnutrition	Normal	0	62.5	0	83.3	0	71.4		
	Moderate	50	12.5	81.8	16.7	70	28.6	0.556	0.407
	Severe	50	25	18.2	0	30	0		

Table S3. Perceived effectiveness of the product depending on treatment (mean ± SD)

		Standard dose of DMB				High dose of DMB				Placebo				P-value		
		Baseline	1 week	2 months	3 months	Baseline	1 week	2 meses	3 meses	Baseline	1 week	2 meses	3 meses	Time (t)	Treatment (T)	T x t
Perceived effectiveness	Points	33.0 ± 27.5	33.0 ± 27.5	62.5 ± 23.8	63.8 ± 21.3	46.5 ± 25.5	44.5 ± 27.2	71.7 ± 24.2	58.3 ± 37.2	66.4 ± 42.9	59.3 ± 40.5	75.0 ± 38.9	75.0 ± 38.9	0.052	0.074	0.628

Table S4. Adverse events depending on the assigned treatment group.

		Standard dose of DMB			High dose of DMB			Placebo			P-value		
		1 month	2 months	3 months	1 month	2 months	3 months	1 month	2 months	3 months	1 month	2 months	3 months
Abdominal distension	Grade 0	90	87.5	100	85.7	66.7	60	100	83.3	83.3			
	Grade 1	10	12.5	0	14.3	16.7	40	0	16.7	16.7	0.649	0.629	0.157
	Grade 2	0	0	0	0	16.7	0	0	0	0			
Abdominal pain	Grade 0	90	100	87.5	85.7	100	60	83.3	100	83.3			
	Grade 1	10	0	12.5	14.3	0	20	16.7	0	16.7	0.578	1	0.520
	Grade 2	0	0	0	0	0	20	0	0	0			
Nausea	Grade 0	90	100	87.5	85.7	83.3	80	100	100	100			
	Grade 1	10	0	12.5	14.3	16.7	20	0	0	0	0.454	0.293	0.545
	Grade 2	0	0	0	0	0	0	0	0	0			
Regurgitation	Grade 0	90	100	75	85.7	100	60	100	83.3	100			
	Grade 1	10	0	25	14.3	0	20	0	16.7	0	0.454	0.293	0.307
	Grade 2	0	0	0	0	0	20	0	0	0			
Vomiting	Grade 0	100	100	100	85.7	83.3	80	83.3	100	100			
	Grade 1	0	0	0	14.3	16.7	20	16.7	0	0	0.426	0.293	0.228
	Grade 2	0	0	0	0	0	0	0	0	0			
Constipation	Grade 0	90	100	87.5	83.3	66.7	40	100	100	66.7			
	Grade 1	10	0	12.5	16.7	33.3	40	0	0	33.3	0.598	0.075	0.301
	Grade 2	0	0	0	0	0	20	0	0	0			
Diarrhea	Grade 0	100	100	100	57.1	100	60	100	100	83.3			
	Grade 1	0	0	0	42.9	0	40	0	0	16.7	0.019	1	0.157
	Grade 2	0	0	0	0	0	0	0	0	0			
Flatulence	Grade 0	70	87.5	75	85.7	50	40	100	100	83.3			
	Grade 1	30	12.5	25	14.3	33.3	40	0	0	16.7	0.158	0.227	0.378
	Grade 2	0	0	0	0	16.7	20	0	0	0			
Intensity	Grade 0	28.6	100	50	40	33.3	50	57.1	83.3	100			
	Grade 1	14.3	0	50	20	0	50	42.9	16.7	0	0.346	0.096	0.247
	Grade 2	57.1	0	0	40	66.7	0	0	0	0			
Product relationship	Grade 0	33.3	100	100	100	100	100	85.7	100	100			
	Grade 1	50	0	0	0	0	0	14.3	0	0	0.118	1	1
	Grade 2	16.7	0	0	0	0	0	0	0	0			
Conduct adopted	Grade 0	33.3	0	0	50	0	0	33.3	0	0			
	Grade 1	50	100	100	50	0	100	66.7	100	100	0.833	0.135	1
	Grade 2	16.7	0	0	0	100	0	0	0	0			

Grade 0, not described; Grade 1, mild; Grade 2, moderate. Values are expressed as percentage.

Table S5. Vitamins and Minerals depending on treatment.

		Standard dose of DMB				High dose of DMB				Placebo				P-value		
		Baseline	1 month	2 months	3 months	Baseline	1 month	2 months	3 months	Baseline	1 month	2 months	3 months	Time (T)	treatment (t)	T x t
Ferritin	ng/mL	137.3±123.9	144.67±181.81	154.25±153.73	331.75±511.44	275.55±297.3	252±309.14	260.76±326.18	216±248.61	1682.4±4770.14	187.14±138.13	208.43±203.11	293.76±294.25	0.215	0.364	0.867
B12	pg/mL	681.4±535.08	721.11±554.97	720±595.86	655.5±353.48	717.73±533.62	891.63±743.36	850.29±642.97	447.67±146.87	816±647.15	647.29±423.41	619.57±270.19	565.86±173.66	0.895	0.79	0.831
Folate	ng/mL	14.45±6.57	14.41±6.92	15.58±6.33	15.79±6.47	15.92±5.95	16.69±7.57	14.95±7.05	15.43±8.67	19.21±7.05	18.67±7.52	16.9±7.9	17.95±8.09	0.139	0.517	0.434
Vitamin A	µg/mL	0.68±0.28	0.59±0.24	0.56±0.23	0.59±0.26	0.67±0.32	0.57±0.24	0.64±0.23	0.73±0.31	0.53±0.12	0.46±0.1	0.51±0.15	0.58±0.18	0.308	0.588	0.362
Vitamin A RBP ratio		1.12±0.11	1.06±0.17	1.06±0.17	1.16±0.11	1.17±0.41	1.04±0.16	1.06±0.12	1.12±0.04	1.11±0.14	1.09±0.07	1.11±0.15	1.16±0.11	0.763	0.522	0.914
Vitamin E	µg/mL	15.7±5.59	13.87±5.39	14.04±6.47	13.89±3.31	14.88±4.25	14.14±5.32	14.07±4.23	14.46±4.67	14.73±3.46	12.37±2.39	14.13±1.62	15.11±3.69	0.372	0.981	0.632
Vitamin E Cholesterol ratio		8.22±1.82	7.69±1.94	8.03±3.05	7.79±1.44	8.65±2.77	8.23±3.61	7.7±2.69	7.73±2.43	8.24±2.79	7.36±1.55	7.79±1.09	8.13±1.94	0.715	0.968	0.855
Vitamin D	ng/mL	22.4±10.47	29.67±18.91	29.5±22.46	24.38±10.88	19.27±15.14	27.5±17.83	19.48±12.49	20.17±12.89	20±13.56	27.29±20.42	42.71±30.61	37.64±23.65	0.325	0.239	0.081
Calcium	mg/dL	9.57±0.38	9.54±0.41	9.48±0.4	9.43±0.54	9.23±0.24	9.35±0.41	9.41±0.48	9.38±0.3	9.04±0.92	9.27±0.3	9.43±0.17	9.44±0.25	0.086	0.48	0.276
Phosphate	mg/dL	3.96±0.56	3.97±0.44	4.13±0.9	3.53±1.17	3.77±0.55	4±0.56	3.64±0.72	3.98±1.01	3.63±0.77	4.01±0.89	3.94±0.97	4.07±1.11	0.658	0.114	0.145
Magnesium	mg/dL	1.78±0.2	1.79±0.23	1.88±0.22	1.81±0.18	1.8±0.22	1.85±0.15	1.88±0.15	1.97±0.16	2.64±2.54	1.7±0.22	1.66±0.25	1.7±0.23	0.262	0.012	0.028

RBP, Retinol binding protein. Values are expressed as mean ± standard deviation.
